# The Intricacy of the Viral-Human Protein Interaction Networks: Resources, Data, and Analyses

**DOI:** 10.3389/fmicb.2022.849781

**Published:** 2022-04-21

**Authors:** Deeya Saha, Marta Iannuccelli, Christine Brun, Andreas Zanzoni, Luana Licata

**Affiliations:** ^1^Aix-Marseille Univ., Inserm, TAGC, UMR_S1090, Marseille, France; ^2^Department of Biology, University of Rome Tor Vergata, Rome, Italy; ^3^CNRS, Marseille, France

**Keywords:** protein-protein interactions, virus-host protein-protein interaction databases, virus-human interactomes, molecular interaction data standards, SARS-CoV-2, emerging viruses

## Abstract

Viral infections are one of the major causes of human diseases that cause yearly millions of deaths and seriously threaten global health, as we have experienced with the COVID-19 pandemic. Numerous approaches have been adopted to understand viral diseases and develop pharmacological treatments. Among them, the study of virus-host protein-protein interactions is a powerful strategy to comprehend the molecular mechanisms employed by the virus to infect the host cells and to interact with their components. Experimental protein-protein interactions described in the scientific literature have been systematically captured into several molecular interaction databases. These data are organized in structured formats and can be easily downloaded by users to perform further bioinformatic and network studies. Network analysis of available virus-host interactomes allow us to understand how the host interactome is perturbed upon viral infection and what are the key host proteins targeted by the virus and the main cellular pathways that are subverted. In this review, we give an overview of publicly available viral-human protein-protein interactions resources and the community standards, curation rules and adopted ontologies. A description of the main virus-human interactome available is provided, together with the main network analyses that have been performed. We finally discuss the main limitations and future challenges to assess the quality and reliability of protein-protein interaction datasets and resources.

## Introduction

Infectious diseases, including respiratory viral infections, are among the top 10 causes of death worldwide accounting for millions of fatalities every year, especially in low-income countries (World Health Organization, 2020). Moreover, the increasing incidence of (re-)emerging infectious diseases is posing serious global health threats (Jones et al., 2008; Cui et al., 2019; Pierson and Diamond, 2020), as exemplified by the COVID-19 pandemic (Morens and Fauci, 2020).

The development of effective antiviral pharmacological treatments relies on an in-depth understanding of the virus biology and the host response (Eckhardt et al., 2020). In the last decades, protein-protein interaction (PPI) discovery experiments have gained momentum among the different approaches to study viral diseases (de Chassey et al., 2014; Goodacre et al., 2020). Indeed, the systematic mapping of interactions between viral and host proteins can provide a better understanding of the molecular mechanisms of viral infections and identify viral perturbations underlying disease phenotypes, thus suggesting novel potential targets of therapeutic intervention (Cakir et al., 2021).

Over the years, these interaction maps described in the scientific literature have been systematically captured into several publicly available molecular interaction databases (e.g., Guirimand et al., 2015; Calderone et al., 2020; Del Toro et al., 2021; Oughtred et al., 2021). The interaction data is organized in structured formats (Orchard et al., 2007; Porras et al., 2020), that can be easily processed and exploited to perform downstream computational and network analyses (Porras et al., 2020).

In this review, we discuss the state-of-the-art of available PPI resources and in particular those dedicated to viruses and the human host. A brief description of the available datasets is provided along with the developed community standards, curation rules, and strategies, adopted ontologies and controlled vocabularies, quality control procedures and scoring systems. We also give an overview of the largest available viral-human interactomes with a particular focus on the recently generated interaction maps between SARS-CoV-2 and human proteins, as well as those of other (re-)emerging viruses like Zika and Dengue, outlining common and virus-specific interaction and host-cell perturbation patterns.

We discuss how these interaction networks can provide novel mechanistic insights on viral infection biology and can suggest novel pharmacological strategies. Finally, we review the main limitations of molecular interaction resources and datasets and their future challenges.

## Public Resources Collecting Virus-Host Protein-Protein Interaction Data

Virus-host molecular interactions, mostly PPIs, detected from high-throughput studies, together with those identified in hundreds of biochemical and biophysical low-throughput studies, have been gathered in distinct public databases using structured formats (Licata and Orchard, 2016; Goodacre et al., 2020).

These public resources can be divided in: *(i*) primary databases that collect only manually curated molecular interactions extracted from peer-reviewed journals and related to different viruses and their relative hosts, such as MINT (Calderone et al., 2020), IntAct (Del Toro et al., 2021), and BioGRID (Oughtred et al., 2021); (*ii*) metadatabases integrating data from primary resources, such as VirusMentha (Calderone et al., 2015) and APID (Alonso-López et al., 2019); (*iii*) databases combining experimental interaction data with predicted PPIs, such as virusSTRING (Szklarczyk et al., 2021), human-virus PPI database (HVIDB) (Yang et al., 2021) and the pathogen-host interaction search tool PHISTO (Durmuş Tekir et al., 2013); (*iv*) databases, such as VirHostnet3.0 database (Guirimand et al., 2015), which are both primary resources collecting manually annotated PPIs and metadatabases integrating data from other molecular interaction databases; and (*v*) databases collecting information only related to a specific virus-host interactome, such as DenHunt (Karyala et al., 2016) and DenvInt (Dey and Mukhopadhyay, 2017) for the Dengue virus, the HIV-1 Human Interaction Database (Ako-Adjei et al., 2015) and the Hepatitis C Virus Protein Interaction Database (HCVpro) (Kwofie et al., 2011).

Despite the large amount of data accumulated over the years in these resources, the early data collection did not follow common criteria in terms of data curation and standardization. This discrepancy in dataset formats and curation strategies is sometimes the cause of heterogeneous data generation, which is difficult to filter, use and analyze without data loss and a time-consuming scrupulous work by bioinformaticians. With this in mind, several years ago, the Molecular Interaction working group of the HUPO-Proteomics Standards Initiative (HUPO-PSI) has developed standards, tools and Controlled Vocabularies (CVs) that have allowed life science communities to combine and analyze datasets collected and stored in different molecular interaction databases (Kerrien et al., 2007; Deutsch et al., 2017). In 2007, the working group defined the minimum information required for reporting a molecular interaction experiment (MIMIx), which enables the systematic capture and the access to interaction data in different resources (Orchard et al., 2007). Several databases have adopted this standard over the years (e.g., BIOGRID, IntAct, MINT, VirHostNet), thus enabling seamless integration of distinct interaction datasets at the minimum level of interaction details, such as interaction detection and participant detection methods.

**FIGURE 1:**
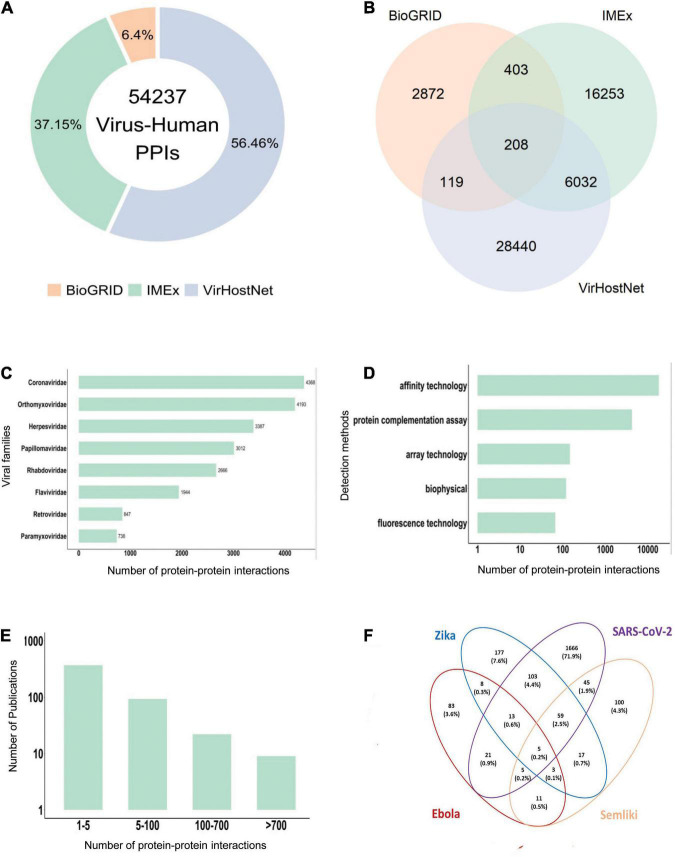
Virus-human protein-protein interaction data statistics. **(A)** Virus-Human PPI data from the three major primary interaction resources (August 2021, BioGRID: 3,943 interactions, IMEx: 22,896 interactions; VirHostNet: 34,799 interactions). **(B)** PPI data overlap among BioGRID, IMEx and VirHostNet databases. **(C)** Number of PPIs in the IMEx dataset for the most representative viral families. **(D)** Number of PPIs in the IMEx dataset according to the experimental methods used for the interaction detection. Methods were grouped in broad categories. For instance, yeast two-hybrid is considered a “protein complementation assay,” and pull-down and coimmunoprecipitation belong to the “affinity technology” category. **(E)** Number of detected PPIs in each paper curated in the IMEx dataset. Most papers describe less than 5 interactions and very few contain more than 100 interactions. The Y-axis is log-transformed. **(F)** Human targets overlap in the PPI network of four emerging viruses. Percentages are computed over the union of all the interactors.

For instance, the integration of virus-human PPIs from the main resources collecting virus-host interactions (e.g., MINT, IntAct, VirHostnet 3.0, and BIOGRID, data fetched in August 2021), generates a very large set of 54,237 interactions between viral and human proteins (Figure 1A). Notably, the overlap between them is very small and mainly consists of the large-scale virus-human interactomes (Figure 1B), suggesting that the different resources may use complementary strategies to mine the available literature.

## International Molecular Exchange Databases, Their Curation Strategies and Adopted Standards

Starting from 2012, some of the major resources collecting PPI data, agreed to unify their curation efforts to obtain a shared and non-redundant dataset, which is annotated using the same curation rules and common export standards.

The result of this coordination is the International Molecular Exchange (IMEx) consortium,^1^ whose members (such as IntAct, MINT, DIP, UniProtKB) have agreed to curate only experimental interaction data coming from peer-reviewed papers.

The consortium members are all professional bio-curators, employing a common detailed curation guideline and up-to-date controlled vocabularies that allow high accuracy of quality control procedures. For instance, interaction data is checked twice before its release, and specific tools are used (e.g., the PSI-MI semantic validator; Montecchi-Palazzi et al., 2009) to automatically check for potential errors and discrepancies related to the PSI-MI ontology of all the entries.

All IMEx entries are annotated with a wealth of details, such as the role played by the participant within the experiment (e.g., bait, prey, neutral), host organism information, cell line or tissue where the experiment was carried out, and several other features related to the interaction, such as binding sites, mutation effect, construct tags, parameters and stoichiometry (Porras et al., 2020).

IMEx datasets can be filtered according to the MIscore, a scoring system that measures the quality of a PPI based on the number of manuscripts reporting the interaction, the type of interaction and the experimental methods used to detect the given interaction (Villaveces et al., 2015).

All IMEx virus-host datasets are available at the IntAct download page.^2^ As of August 2021, the IMEx virus-human dataset consists of 22,896 PPIs (Supplementary Table 1). Coronaviridae, Orthomyxoviridae, Papillomaviridae, and Herpesviridae are among the most represented families (Figure 1C). The datasets contain not only virus-human host interactions, but also interactions involving proteins from other animal viruses and hosts. A dedicated COVID-19 dataset is as well available (Perfetto et al., 2020).

## Main Limitations of Protein-Protein Interaction Data and Their Impact on Interactome Analysis

Studies of virus-host interactomes have turned out to be extremely powerful to identify the main host target proteins and the biological processes perturbed during a viral infection, but also to predict new potential therapeutic targets and drugs repurposing candidates (Bouhaddou et al., 2020; Gordon et al., 2020b).

However, the quality and reliability of these analyses are biased by several factors, such as the interactome coverage, the methodologies applied to generate the PPI network, the lack of low throughput validation studies, true negative PPIs and the types of standards adopted to annotate those data (Braun et al., 2009; Venkatesan et al., 2009).

Most of the available molecular interaction data is associated with the frequently studied viral families due to their impact on public health and global economy (Figure 1C). Their interactomes are often the result of large-scale yeast two-hybrid or AP-MS screens (de Chassey et al., 2008; Shapira et al., 2009; Tripathi et al., 2010; Muller et al., 2012; Dolan et al., 2013; Wang et al., 2017; Gordon et al., 2020a; Li et al., 2021; Stukalov et al., 2021; Supplementary Table 2 and Figure 1D).

According to the methodology applied, different subsets of PPIs and different interaction types (direct or indirect) can be detected, and this partially explains the poor overlap often observed between large-scale PPI datasets (Braun, 2012). Furthermore, these differences are often related to the strategies employed by researchers during the selection of high confident interactors and the removal of spurious interactors (Walhout and Vidal, 1999; Hein et al., 2015; Choi et al., 2019).

As an example, the three main high-throughput experimental screens to map the interactome between SARS-CoV-2 and human proteins employed similar AP-MS methodologies (Gordon et al., 2020a; Li et al., 2021; Stukalov et al., 2021). However, Gordon et al., 2020a and Li et al. (2021) used HEK293T cells, while Stukalov et al. (2021) used A549 cells. Despite the use of the same technique and in two cases of the same cell line, the three screens detected a different number of interactions and showed a poor overlap in terms of human targets. However, pathway enrichment analyses revealed commonalities in the biological processes and cellular pathways targeted by viral proteins, such as cell cycle and response to stress (Perfetto et al., 2020).

This variability can be further amplified by different experimental conditions, tissues or cell lines used or experimental and participant modifications (e.g., use of chemicals or drugs, use and position of a tag, protein mutations).

Ammari et al. (2018) showed that the use of rich datasets, such as the ones provided by IMEx resources, allows performing more comprehensive network analysis whose output can differ greatly depending on the biological context or methodology used. For example, the host interacting partners of HCV proteins change depending on the cell line used to perform the experiments (e.g., Huh7 vs. HEK293) and consequently the cellular processes in which they are involved (Ammari et al., 2018).

All these aspects must be considered before selecting, merging and analyzing PPI datasets. The choice of a dataset containing information on the biological context (Porras et al., 2020) in which the interactions have occurred, can allow more sophisticated analysis and reliable outcomes.

Another important aspect that can strongly impact the evaluation of the quality of a virus-host interactome is the use of small-scale biochemical and biophysical studies that can validate and confirm the interactions found in large-scale experiments. A detailed analysis of the available validated virus-host interactions has been presented in a recent review (Goodacre et al., 2020).

*In-silico* approaches based on sequence (e.g., Eid et al., 2016; Liu-Wei et al., 2021) and structural similarity (e.g., de Chassey et al., 2013; Lasso et al., 2019), as well as protein docking (Wierbowski et al., 2021), have been also used to predict virus-host protein-protein interactions. The recent advent of deep-learning methods to predict protein structures (Senior et al., 2020; Baek et al., 2021) as well as protein macromolecular complexes (Baek et al., 2021; Bryant et al., 2021; Evans et al., 2021), can be a useful complementary strategy to identify or validate the molecular determinants of virus-host protein interactions identified in experimental assays.

Finally, negative PPIs can be extremely important for validating interaction data or to assess the quality of interaction prediction methods. To our knowledge, the Negatome Database 2.0 is the only available resource collecting valuable negative interaction data (Blohm et al., 2014). Indeed, the database lists experimentally verified non-interacting proteins identified either by manual curation from literature (2,171 negative interactions, 75 of which involve at least one viral protein) or derived by the analysis of the protein structures from the PDB (4,397 negative interactions, only two involve at least one viral protein).

The IMEx consortium databases also collect negative interactions (Porras et al., 2020). However, the size of the dataset is still small (∼1,000 PPIs) and only 18 of those are negative virus-host interactions, suggesting that, on one hand, researchers should systematically provide the negative interaction data coming from their experiments, and on the other hand, additional curation effort is needed to extract this information from the scientific literature.

## Viral-Human Interactomes: From Network Perturbation to Dysregulated Biological Processes in Disease

Over the past two decades, several high-throughput techniques, such as yeast two-hybrid and affinity purification coupled to mass spectrometry (AP-MS), have been developed to map model organism interactomes in order to decipher the dynamics and complexity of interaction networks (Snider et al., 2015). These methodologies have also been applied to chart the interactome between several viruses and the human host (Supplementary Table 2 and Figures 1D,E).

The first virus-host interaction maps that have been deciphered (EBV, HCV) revealed that viral proteins preferentially target highly connected proteins (hubs) among their host proteins (Calderwood et al., 2007; de Chassey et al., 2008). As these hub proteins are relatively close in the network to a large number of proteins involved in different cellular processes, this could represent a virus strategy to subvert the cellular processes at its own benefit (Bösl et al., 2019).

Early structural bioinformatics analyses showed that human-cell hijacking by viral proteins can be achieved through interface mimicry of endogenous interactions (i.e., interaction between host proteins) (Franzosa and Xia, 2011; Garamszegi et al., 2013). Notably, they estimated that up to one-third of the viral-human interactions studied can be related to this phenomenon, in particular through the mimicry of non-globular protein interaction elements known as short linear motifs (SLiMs), which are short stretches of contiguous amino acids residues that often mediate transient PPIs (Davey et al., 2012) and have emerged through convergent evolution (Davey et al., 2011). Viral abuse of SLiMs is widespread (Davey et al., 2011; Hagai et al., 2014; Via et al., 2015), and the pervasiveness of interface mimicry provides potential connections between infectious agents and human diseases (Chen and Xia, 2019; Lasso et al., 2021).

Indeed, the targeted and consequently perturbed processes by human viruses encompass different and relevant signaling pathways: TGFbeta for SARS-CoV-2 and Hepatitis C Virus (HCV) (de Chassey et al., 2008; Stukalov et al., 2021); JAK/STAT for HCV (de Chassey et al., 2008); Notch for Epstein-Barr Virus (EBV), Human Papillomavirus (HPV), Polyoma Virus (PyV), and Adenovirus (Ad5) (Fossum et al., 2009); Wnt for Influenza A Virus (IAV-H1N1) (Shapira et al., 2009), and cellular processes such as autophagy (SARS-CoV-2) (Stukalov et al., 2021), apoptosis (EBV, HPV, PyV, and Ad5) (Fossum et al., 2009), focal adhesion (HCV) (de Chassey et al., 2008) or non-sense-mediated mRNA decay [Semliki Forest Virus (SFV); Contu et al., 2021]. The identification of targeted cellular functions is usually performed using computational tools for functional enrichment analysis such as g:Profiler (Raudvere et al., 2019) and Metascape (Zhou et al., 2019).

The blockade of some key factors through interactions is also often observed from PPI analysis. Whereas SARS-CoV-2 proteins perturb the NF-kB-repressing factor (NKRF), therefore potentially contributing to the host inflammatory response by acting on the IL-8-mediated chemotaxis of neutrophils (Li et al., 2021), the Ebola virus increases its own transcription and replication by interfering with an ubiquitin ligase (RBBP6) (Batra et al., 2018). Zika and Dengue viruses suppresses interferon-stimulated genes by inhibiting the recruitment of the transcription complex PAF1C (Shah et al., 2018), and HIV protects its replication by cleaving EIF3D, a subunit of eukaryotic translation initiation factor 3, able to inhibit HIV replication (Jäger et al., 2012). Conversely, interactome analysis also allows discovering host proteins that protect against infection such as Plakophilin 2 (PKP2), a natural inhibitor of IAV polymerase complex (Wang et al., 2017).

In addition, interaction analysis can explain disease phenotypes and unravel pathogenic mechanisms. The Zika virus (ZIKV) can cause neurodevelopmental defects (Platt et al., 2018). The viral NS4A protein interacts with a gene linked to hereditary microcephaly in humans (hANKLE2) (Shah et al., 2018). Strikingly, the ubiquitous expression of NS4A in wild type Drosophila phenocopies microcephaly that, in turn, is rescued by the expression of hANKLE2 and or its ortholog in Drosophila (dAnkle2) (Shah et al., 2018). Virus-host PPI mapping therefore provides biological insights and unveils potential pathogenic mechanisms.

Finally, although beyond the scope of this review, in the case of vector-borne diseases such as Dengue and Zika fever, the comparison between the virus-vector and the virus-host interaction maps (i.e., Shah et al., 2018) can reveal promising drug target candidates or treatment strategies to reduce the risk of viral resistance.

## Viral-Host Interactomes of the Emerging Viruses: Commonalities and Specificities

Viruses have evolved sophisticated strategies to enter and evade host-cell defense and accelerate viral replication by perturbing a variety of cellular functions. Several integrated network analyses revealed that some of these strategies are virus-specific whereas others perturb common cellular pathways (Pichlmair et al., 2012; Shah et al., 2018; Bösl et al., 2019).

In this section, we focus on four emerging viruses (SARS-CoV-2, Ebola virus, ZIKV, and SFV), for which a repertoire of PPIs with human proteins in the IMEx consortium databases is available. As shown previously (Bösl et al., 2019), the four viruses show both common and specific human protein interactors (Figure 1F) as well as targeted biological processes. For instance, among the commonly targeted cellular functions, the most represented are related to protein translation and RNA processing (Supplementary Table 3), in agreement with the biology of RNA viruses. Indeed, around one quarter of the known ∼2,000 human RNA binding proteins (RBPs) has been shown to play a critical role during viral infection (Garcia-Moreno et al., 2018).

Interestingly, only five human interactors are shared by all the four viruses, and four out of five are RNA binding proteins or RBPs. One of them is the prohibitin (PHB1), which is known for its role in cell-to-cell transmission of herpes virus (Watanabe et al., 2021) and plays a pivotal role during other viral infections like that of Enterovirus and HCV (Liu et al., 2015; Too et al., 2018). Interestingly, RBPs that are commonly targeted by ZIKV, SFV, and SARS-CoV-2 are not only involved in mRNA translation but in many other immunoregulatory processes. Fifty-nine proteins are commonly targeted by ZIKV, SFV and SARS-CoV-2 (Figure 1F). All of them have RNA binding activity and some of them also take active part in immune regulation. For instance, DDX21, an RNA helicase, acts in innate immune response as positive regulator of NF-kB signaling (Zhang et al., 2011; Chen et al., 2014; Abdullah et al., 2021) and as antiviral factor (Chen et al., 2014). In addition, many RBPs commonly targeted by the three viruses are associated with ubiquitin mediated protein degradation pathways (e.g., RPS7, RPL11, RPS2, RPL5) and regulation of apoptotic processes (e.g., SERBP1, RSL1D1, RPS7), thus underlying the key role of RBPs in virus-host interactions. Do these emerging viruses strategically target RBPs, as also shown for IAV-H1N1 (Shapira et al., 2009)? If this is the case, what are the consequences of the hijacking of RBPs on host defense response upon infection? These are still open questions. However, recent studies highlight the antiviral or immune related function of RBPs (Newman et al., 2015; Díaz-Muñoz and Turner, 2018; Garcia-Moreno et al., 2019) and their implication in viral processes (Embarc-Buh et al., 2021; Kamel et al., 2021).

Among SARS-CoV-2 specific human targets, there are 23 proteins linked to ER-associated protein degradation pathways members, such as BAG6 and STUB1. Recently, a study has shown that ER stress inducer thapsigargin inhibits coronavirus replication (Shaban et al., 2021). Moreover, coronaviruses, including SARS-CoV-2, suppress ER quality control processes or ER associated degradation which is re-activated by the drug thapsigargin (Shaban et al., 2021). Hence, targeting of ER-associated degradation pathways (ERAD) pathways by SARS-CoV-2 or other coronaviruses could be a unique strategy to evade host defense and facilitate viral replication within the host.

ZIKV specific human targets are mainly involved in mitochondrial translation. Recent studies show that ZIKV infection impairs mitochondrial functions (Yang et al., 2020; Yau et al., 2021). On the other hand, SFV specific interactors are involved in non-sense mediated mRNA decay (NMD) (Contu et al., 2021). Indeed, SFV inhibits NMD, which in turn helps the stabilization of the viral genomic RNA within the host cell (Contu et al., 2021).

Altogether, a quick scrutiny of the human interactors of these four emerging viruses sheds light on some of the common as well as specific strategies to subvert host cellular machinery. Further and deeper investigation of these common and specific human proteins can therefore generate testable hypotheses on the infection biology of emerging and re-emerging diseases.

## Conclusion and Future Challenges

PPI databases are important resources to gather and organize in structured formats virus-host PPI datasets useful for further network analysis. A better coverage of the curated virus-host PPIs together with the complete annotation of the experimental feature details, such as the biological context of an interaction, are necessary to perform more sophisticated network analysis. Indeed, network analysis has been proved to be fundamental to understand the perturbed cellular machinery by viruses.

Reverse genetic systems are used to manipulate virus genomes in order to understand genotypic variation or to investigate specific gene functions (Messer et al., 2012; V’kovski et al., 2021). These technologies can be also useful to contextualize virus-host PPIs during the virus life cycle and to gain important information on virus pathological processes at the molecular level.

Furthermore, the integration of interactome data with available proteomic, genetic, structural and clinical data can give a more comprehensive picture of the biological process perturbed during viral infection, paving the way to the identification of novel drug targets and successful treatments (Bouhaddou et al., 2020; Gordon et al., 2020b; Wierbowski et al., 2021).

## Author Contributions

AZ and LL wrote the first draft of the manuscript. DS, MI, and CB wrote sections of the manuscript. DS prepared the figure. All authors contributed to manuscript revision, read, and approved the submitted version.

## Conflict of Interest

The authors declare that the research was conducted in the absence of any commercial or financial relationships that could be construed as a potential conflict of interest.

## Publisher’s Note

All claims expressed in this article are solely those of the authors and do not necessarily represent those of their affiliated organizations, or those of the publisher, the editors and the reviewers. Any product that may be evaluated in this article, or claim that may be made by its manufacturer, is not guaranteed or endorsed by the publisher.

## Footnotes

1
http://www.imexconsortium.org/

2
https://www.ebi.ac.uk/intact/download/datasets
