# Supplementary material for: The Intricacy of the Viral-Human Protein Interaction Networks: Resources, Data, and Analyses
Source: Front Microbiol. 2022 Apr 21;13:849781. doi: 10.3389/fmicb.2022.849781 (PMC9069133; doi:10.3389/fmicb.2022.849781)
Supplement: Supplementary file 1 [file Data_Sheet_1.docx]

**Supplementary Information**

**The intricacy of the viral-human protein interaction networks: resources, data and analyses**

Deeya Saha^1^, Marta Iannuccelli^2^, Christine Brun^1,3^, Andreas Zanzoni^1^, Luana Licata^2^

^1^ Aix-Marseille Univ, Inserm, TAGC UMR_S1090, Marseille, France. ^2^ Department of Biology, University of Rome Tor Vergata, Rome, Italy. ^3^ CNRS, Marseille, France.

**Supplementary Tables**

**Supplementary Table 1.** Number of interactions with human proteins for each viral family curated in IMEx consortium databases as of August 2021. Only interaction data coming from articles published in peer-reviewed journals is taken into account.

| **Viral family** | **Number of protein-protein interactions** | **Baltimore classification** |
| --- | --- | --- |
| Coronaviridae | 4368 | +ssRNA |
| Orthomyxoviridae | 4193 | -ssRNA |
| Herpesviridae | 3387 | dsDNA |
| Papillomaviridae | 3012 | dsDNA |
| Rhabdoviridae | 2666 | -ssRNA |
| Flaviviridae | 1944 | +ssRNA |
| Retroviridae | 847 | rtRNA |
| Paramyxoviridae | 738 | -ssRNA |
| Pneumoviridae | 305 | -ssRNA |
| Adenoviridae | 209 | dsDNA |
| Poxviridae | 193 | dsDNA |
| Filoviridae | 179 | -ssRNA |
| Phenuiviridae | 124 | -ssRNA |
| Polyomaviridae | 117 | dsDNA |
| Peribunyaviridae | 83 | -ssRNA |
| Matonaviridae | 62 | +ssRNA |
| Reoviridae | 58 | dsRNA |
| Arteriviridae | 56 | +ssRNA |
| Hepadnaviridae | 31 | rtDNA |
| Arenaviridae | 19 | -ssRNA |
| Picornaviridae | 15 | +ssRNA |
| Parvoviridae | 9 | ssDNA |
| Asfarviridae | 5 | dsDNA |
| Nairoviridae | 2 | -ssRNA |
| Microviridae | 1 | ssDNA |
| *Unassigned* | *273* | *-* |

**Supplementary Table 2**. Selection of virus-human high-throughput interaction screens curated in primary databases and published in peer-reviewed journals as August 2021. Only screens with more than 100 interactions were selected.

| **Virus name** | **Number of interactions** | **Number of viral proteins** | **Number of human proteins** | **Interaction method** | **Reference(s)** |
| --- | --- | --- | --- | --- | --- |
| Dengue virus (DENV) | 139 | 10 | 105 | two hybrid | (Khadka et al., 2011) |
| Dengue virus (DENV) | 498 | 10 | 189 | affinity chromatography technology | (Shah et al., 2018) |
| Ebola virus (EBOV) | 195 | 6 | 170 | affinity chromatography technology | (Batra et al., 2018) |
| Epstein-Barr virus (EBV) | 173 | 40 | 112 | two hybrid | (Calderwood et al., 2007) |
| Hepatitis C virus (HCV) | 314 | 11 | 278 | two hybrid | (de Chassey et al., 2008) |
| Hepatitis C virus (HCV) | 112 | 7 | 94 | two hybrid | (Dolan et al., 2013) |
| Influenza A virus (IAV, H1N1) | 215 | 10 | 99 | two hybrid | (Shapira et al., 2009) |
| Influenza A virus (IAV, H1N1) | 529 | 11 | 357 | anti tag coimmunoprecipitation | (Wang et al., 2017) |
| Human immunodeficiency virus (HIV) | 497 | 18 | 435 | affinity chromatography technology | (Jäger et al., 2011) |
| Middle East respiratory syndrome coronavirus (MERS-CoV) | 296 | 22 | 292 | pull down | (Gordon et al., 2020a) |
| Severe acute respiratory syndrome coronavirus (SARS-CoV) | 366 | 25 | 365 | pull down | (Gordon et al., 2020a) |
| Severe acute respiratory syndrome coronavirus (SARS-CoV) | 713 | 27 | 624 | anti tag coimmunoprecipitation | (Stukalov et al., 2021) |
| Severe acute respiratory syndrome coronavirus 2 (SARS-CoV-2) | 389 | 27 | 384 | pull down | (Gordon et al., 2020b, 2020a) |
| Severe acute respiratory syndrome coronavirus 2 (SARS-CoV-2) | 1092 | 24 | 877 | anti tag coimmunoprecipitation | (Stukalov et al., 2021) |
| Severe acute respiratory syndrome coronavirus 2 (SARS-CoV-2) | 295 | 19 | 286 | anti tag coimmunoprecipitation | (Li et al., 2021) |
| Semliki forest virus (SFV) | 337 | 6 | 244 | anti tag coimmunoprecipitation | (Contu et al., 2021) |
| Zika virus (ZIKV) | 368 | 5 | 365 | affinity chromatography technology | (Shah et al., 2018) |

**Supplementary Table 3**. Functional enrichment analysis of the human targets of four emerging viruses. The analysis was performed with the g:Profiler webtool (<https://biit.cs.ut.ee/gprofiler/gost>, version e105_eg52_p16_e84549f) using the following parameters: Gene Ontology Biological Process (BP) terms as annotation source; terms with less of 5 or more than 500 annotated genes were discarded; adjusted p-value threshold = 0.01; statistical background: the annotated human genome. The list of human interactors was gathered from the publications of four large-scale interaction discovery experiments performed with affinity-based techniques and listed in Supplementary Table 2. EBOV: Ebola virus (Batra et al., 2018); SARS2_GORDON: SARS-CoV-2 interaction data from (Gordon et al., 2020a, 2020b); SARS2_LI: SARS-CoV-2 interaction data from (Li et al., 2021); SARS2_STUKALOV: SARS-CoV-2 interaction data from (Stukalov et al., 2021); SFV: Semliki virus (Contu et al., 2021); ZIKV: Zika virus (Shah et al., 2018).

| **Virus** | **Type** | **Term name** | **Term id** | **Adjusted**  **p-value** |
| --- | --- | --- | --- | --- |
| EBOV | BP | rRNA metabolic process | GO:0016072 | 2.17E-18 |
| EBOV | BP | rRNA processing | GO:0006364 | 7.34E-14 |
| EBOV | BP | ribosome biogenesis | GO:0042254 | 1.92E-13 |
| EBOV | BP | ncRNA processing | GO:0034470 | 7.95E-13 |
| EBOV | BP | regulation of RNA splicing | GO:0043484 | 2.62E-07 |
| EBOV | BP | regulation of mRNA processing | GO:0050684 | 1.5806E-06 |
| EBOV | BP | RNA 3'-end processing | GO:0031123 | 6.27151E-06 |
| EBOV | BP | rRNA transcription | GO:0009303 | 1.12135E-05 |
| EBOV | BP | regulation of mRNA splicing, via spliceosome | GO:0048024 | 1.43167E-05 |
| EBOV | BP | regulation of mRNA metabolic process | GO:1903311 | 4.07279E-05 |
| EBOV | BP | regulation of alternative mRNA splicing, via spliceosome | GO:0000381 | 4.28071E-05 |
| EBOV | BP | mRNA polyadenylation | GO:0006378 | 0.000248823 |
| EBOV | BP | RNA polyadenylation | GO:0043631 | 0.000284879 |
| EBOV | BP | alternative mRNA splicing, via spliceosome | GO:0000380 | 0.000296305 |
| EBOV | BP | mitochondrial translation | GO:0032543 | 0.000296305 |
| EBOV | BP | ncRNA transcription | GO:0098781 | 0.00047641 |
| EBOV | BP | RNA phosphodiester bond hydrolysis | GO:0090501 | 0.000589355 |
| EBOV | BP | RNA catabolic process | GO:0006401 | 0.001319962 |
| EBOV | BP | nuclear transport | GO:0051169 | 0.001472075 |
| EBOV | BP | nucleocytoplasmic transport | GO:0006913 | 0.001472075 |
| EBOV | BP | nucleic acid phosphodiester bond hydrolysis | GO:0090305 | 0.002272788 |
| EBOV | BP | mRNA 3'-end processing | GO:0031124 | 0.00265347 |
| EBOV | BP | sno(s)RNA 3'-end processing | GO:0031126 | 0.002971848 |
| EBOV | BP | mitochondrial gene expression | GO:0140053 | 0.004088317 |
| EBOV | BP | nuclear-transcribed mRNA catabolic process | GO:0000956 | 0.004374815 |
| EBOV | BP | sno(s)RNA processing | GO:0043144 | 0.005939184 |
| EBOV | BP | viral process | GO:0016032 | 0.007642033 |
| SARS2_GORDON | BP | establishment of protein localization to mitochondrion | GO:0072655 | 1.19514E-05 |
| SARS2_GORDON | BP | protein localization to mitochondrion | GO:0070585 | 2.06882E-05 |
| SARS2_GORDON | BP | protein insertion into mitochondrial inner membrane | GO:0045039 | 5.56124E-05 |
| SARS2_GORDON | BP | vesicle-mediated transport to the plasma membrane | GO:0098876 | 6.98916E-05 |
| SARS2_GORDON | BP | endocytic recycling | GO:0032456 | 0.000156296 |
| SARS2_GORDON | BP | ERAD pathway | GO:0036503 | 0.000167597 |
| SARS2_GORDON | BP | establishment of protein localization to mitochondrial membrane | GO:0090151 | 0.000240585 |
| SARS2_GORDON | BP | protein targeting to mitochondrion | GO:0006626 | 0.000450814 |
| SARS2_GORDON | BP | inner mitochondrial membrane organization | GO:0007007 | 0.000754828 |
| SARS2_GORDON | BP | endosomal transport | GO:0016197 | 0.001856567 |
| SARS2_GORDON | BP | mitochondrial transport | GO:0006839 | 0.002324414 |
| SARS2_GORDON | BP | protein folding | GO:0006457 | 0.003177385 |
| SARS2_GORDON | BP | snRNA metabolic process | GO:0016073 | 0.003490133 |
| SARS2_GORDON | BP | snRNA processing | GO:0016180 | 0.005019727 |
| SARS2_GORDON | BP | Golgi vesicle transport | GO:0048193 | 0.006015461 |
| SARS2_GORDON | BP | establishment of protein localization to membrane | GO:0090150 | 0.006579505 |
| SARS2_GORDON | BP | mRNA transport | GO:0051028 | 0.007102124 |
| SARS2_GORDON | BP | DNA replication, synthesis of RNA primer | GO:0006269 | 0.007271143 |
| SARS2_GORDON | BP | Golgi organization | GO:0007030 | 0.007651963 |
| SARS2_GORDON | BP | response to endoplasmic reticulum stress | GO:0034976 | 0.007995398 |
| SARS2_GORDON | BP | positive regulation of cellular catabolic process | GO:0031331 | 0.008103557 |
| SARS2_GORDON | BP | I-kappaB kinase/NF-kappaB signaling | GO:0007249 | 0.008756456 |
| SARS2_GORDON | BP | glycoprotein metabolic process | GO:0009100 | 0.009408108 |
| SARS2_Li | BP | response to endoplasmic reticulum stress | GO:0034976 | 4.01E-07 |
| SARS2_Li | BP | protein folding | GO:0006457 | 7.13442E-06 |
| SARS2_Li | BP | establishment of RNA localization | GO:0051236 | 9.87139E-06 |
| SARS2_Li | BP | RNA localization | GO:0006403 | 1.64399E-05 |
| SARS2_Li | BP | protein N-linked glycosylation via asparagine | GO:0018279 | 2.74213E-05 |
| SARS2_Li | BP | peptidyl-asparagine modification | GO:0018196 | 3.82864E-05 |
| SARS2_Li | BP | ERAD pathway | GO:0036503 | 4.68052E-05 |
| SARS2_Li | BP | response to topologically incorrect protein | GO:0035966 | 4.93692E-05 |
| SARS2_Li | BP | nucleic acid transport | GO:0050657 | 6.26611E-05 |
| SARS2_Li | BP | RNA transport | GO:0050658 | 6.26611E-05 |
| SARS2_Li | BP | protein hydroxylation | GO:0018126 | 0.00012531 |
| SARS2_Li | BP | nuclear export | GO:0051168 | 0.000261882 |
| SARS2_Li | BP | mRNA transport | GO:0051028 | 0.000280064 |
| SARS2_Li | BP | protein localization to centrosome | GO:0071539 | 0.000427808 |
| SARS2_Li | BP | ubiquitin-dependent ERAD pathway | GO:0030433 | 0.0004409 |
| SARS2_Li | BP | response to unfolded protein | GO:0006986 | 0.000588023 |
| SARS2_Li | BP | protein localization to microtubule organizing center | GO:1905508 | 0.000658682 |
| SARS2_Li | BP | protein localization to microtubule cytoskeleton | GO:0072698 | 0.001046367 |
| SARS2_Li | BP | Golgi vesicle transport | GO:0048193 | 0.0014416 |
| SARS2_Li | BP | nuclear transport | GO:0051169 | 0.001649307 |
| SARS2_Li | BP | nucleocytoplasmic transport | GO:0006913 | 0.001649307 |
| SARS2_Li | BP | protein localization to cytoskeleton | GO:0044380 | 0.001843438 |
| SARS2_Li | BP | RNA export from nucleus | GO:0006405 | 0.002716925 |
| SARS2_Li | BP | peptidyl-proline modification | GO:0018208 | 0.003109675 |
| SARS2_Li | BP | nucleobase-containing compound transport | GO:0015931 | 0.003561371 |
| SARS2_Li | BP | establishment of organelle localization | GO:0051656 | 0.004209895 |
| SARS2_Li | BP | vesicle-mediated transport to the plasma membrane | GO:0098876 | 0.00549657 |
| SARS2_Li | BP | peptidyl-lysine hydroxylation | GO:0017185 | 0.007805225 |
| SARS2_Li | BP | ATP metabolic process | GO:0046034 | 0.008080363 |
| SARS2_Li | BP | mitochondrial ATP synthesis coupled proton transport | GO:0042776 | 0.008328785 |
| SARS2_STUKALOV | BP | Golgi vesicle transport | GO:0048193 | 2.15E-17 |
| SARS2_STUKALOV | BP | glycoprotein biosynthetic process | GO:0009101 | 5.76E-16 |
| SARS2_STUKALOV | BP | Golgi organization | GO:0007030 | 6.39E-14 |
| SARS2_STUKALOV | BP | glycoprotein metabolic process | GO:0009100 | 2.35E-13 |
| SARS2_STUKALOV | BP | macromolecule glycosylation | GO:0043413 | 7.61E-13 |
| SARS2_STUKALOV | BP | protein glycosylation | GO:0006486 | 7.61E-13 |
| SARS2_STUKALOV | BP | glycosylation | GO:0070085 | 6.19E-12 |
| SARS2_STUKALOV | BP | organelle fusion | GO:0048284 | 1.35E-11 |
| SARS2_STUKALOV | BP | vesicle organization | GO:0016050 | 2.16E-11 |
| SARS2_STUKALOV | BP | vesicle fusion | GO:0006906 | 2.21E-11 |
| SARS2_STUKALOV | BP | organelle membrane fusion | GO:0090174 | 5.79E-11 |
| SARS2_STUKALOV | BP | endoplasmic reticulum to Golgi vesicle-mediated transport | GO:0006888 | 1.01E-09 |
| SARS2_STUKALOV | BP | membrane docking | GO:0022406 | 1.21E-09 |
| SARS2_STUKALOV | BP | membrane fusion | GO:0061025 | 1.32E-09 |
| SARS2_STUKALOV | BP | organelle localization by membrane tethering | GO:0140056 | 9.16E-08 |
| SARS2_STUKALOV | BP | extrinsic apoptotic signaling pathway | GO:0097191 | 1.78E-07 |
| SARS2_STUKALOV | BP | vesicle docking | GO:0048278 | 6.83E-07 |
| SARS2_STUKALOV | BP | protein N-linked glycosylation | GO:0006487 | 2.02912E-06 |
| SARS2_STUKALOV | BP | gland development | GO:0048732 | 2.45794E-06 |
| SARS2_STUKALOV | BP | lipoprotein biosynthetic process | GO:0042158 | 5.17731E-06 |
| SARS2_STUKALOV | BP | cytosolic transport | GO:0016482 | 6.8143E-06 |
| SARS2_STUKALOV | BP | intra-Golgi vesicle-mediated transport | GO:0006891 | 8.26434E-06 |
| SARS2_STUKALOV | BP | protein lipidation | GO:0006497 | 1.37233E-05 |
| SARS2_STUKALOV | BP | response to endoplasmic reticulum stress | GO:0034976 | 2.30439E-05 |
| SARS2_STUKALOV | BP | iron ion homeostasis | GO:0055072 | 3.02464E-05 |
| SARS2_STUKALOV | BP | ubiquitin-dependent ERAD pathway | GO:0030433 | 4.36721E-05 |
| SARS2_STUKALOV | BP | transmembrane receptor protein serine/threonine kinase signaling pathway | GO:0007178 | 4.81224E-05 |
| SARS2_STUKALOV | BP | protein refolding | GO:0042026 | 5.81057E-05 |
| SARS2_STUKALOV | BP | lipoprotein metabolic process | GO:0042157 | 6.00759E-05 |
| SARS2_STUKALOV | BP | endoplasmic reticulum organization | GO:0007029 | 8.81356E-05 |
| SARS2_STUKALOV | BP | regulation of transmembrane receptor protein serine/threonine kinase signaling pathway | GO:0090092 | 0.000249091 |
| SARS2_STUKALOV | BP | ERAD pathway | GO:0036503 | 0.000296715 |
| SARS2_STUKALOV | BP | proteoglycan biosynthetic process | GO:0030166 | 0.00029735 |
| SARS2_STUKALOV | BP | protein palmitoylation | GO:0018345 | 0.000465114 |
| SARS2_STUKALOV | BP | transition metal ion homeostasis | GO:0055076 | 0.000479943 |
| SARS2_STUKALOV | BP | retrograde transport, endosome to Golgi | GO:0042147 | 0.000527566 |
| SARS2_STUKALOV | BP | positive regulation of protein catabolic process | GO:0045732 | 0.000599021 |
| SARS2_STUKALOV | BP | wound healing | GO:0042060 | 0.000906405 |
| SARS2_STUKALOV | BP | ameboidal-type cell migration | GO:0001667 | 0.000962569 |
| SARS2_STUKALOV | BP | cell growth | GO:0016049 | 0.000963761 |
| SARS2_STUKALOV | BP | regulation of protein catabolic process | GO:0042176 | 0.001065599 |
| SARS2_STUKALOV | BP | response to oxygen levels | GO:0070482 | 0.001245715 |
| SARS2_STUKALOV | BP | regulation of body fluid levels | GO:0050878 | 0.001249334 |
| SARS2_STUKALOV | BP | vacuole organization | GO:0007033 | 0.001386179 |
| SARS2_STUKALOV | BP | response to oxidative stress | GO:0006979 | 0.001456467 |
| SARS2_STUKALOV | BP | cellular iron ion homeostasis | GO:0006879 | 0.001511327 |
| SARS2_STUKALOV | BP | cellular response to unfolded protein | GO:0034620 | 0.001730654 |
| SARS2_STUKALOV | BP | protein N-linked glycosylation via asparagine | GO:0018279 | 0.002368083 |
| SARS2_STUKALOV | BP | protein localization to plasma membrane | GO:0072659 | 0.002582757 |
| SARS2_STUKALOV | BP | regulation of extrinsic apoptotic signaling pathway | GO:2001236 | 0.002582941 |
| SARS2_STUKALOV | BP | viral process | GO:0016032 | 0.003071527 |
| SARS2_STUKALOV | BP | peptidyl-asparagine modification | GO:0018196 | 0.003435373 |
| SARS2_STUKALOV | BP | cellular response to topologically incorrect protein | GO:0035967 | 0.004155097 |
| SARS2_STUKALOV | BP | membrane lipid biosynthetic process | GO:0046467 | 0.004570688 |
| SARS2_STUKALOV | BP | endosomal transport | GO:0016197 | 0.004575438 |
| SARS2_STUKALOV | BP | response to topologically incorrect protein | GO:0035966 | 0.004749186 |
| SARS2_STUKALOV | BP | sulfur compound metabolic process | GO:0006790 | 0.004923762 |
| SARS2_STUKALOV | BP | amyloid precursor protein metabolic process | GO:0042982 | 0.005121616 |
| SARS2_STUKALOV | BP | viral entry into host cell | GO:0046718 | 0.005624549 |
| SARS2_STUKALOV | BP | regulation of cell growth | GO:0001558 | 0.007537484 |
| SARS2_STUKALOV | BP | positive regulation of cardiac epithelial to mesenchymal transition | GO:0062043 | 0.007844061 |
| SARS2_STUKALOV | BP | cellular response to increased oxygen levels | GO:0036295 | 0.007863316 |
| SARS2_STUKALOV | BP | fatty acid metabolic process | GO:0006631 | 0.008124168 |
| SARS2_STUKALOV | BP | movement in host environment | GO:0052126 | 0.008454891 |
| SARS2_STUKALOV | BP | response to unfolded protein | GO:0006986 | 0.008984132 |
| SARS2_STUKALOV | BP | unsaturated fatty acid metabolic process | GO:0033559 | 0.009248514 |
| SARS2_STUKALOV | BP | protein localization to cell periphery | GO:1990778 | 0.009433797 |
| SFV | BP | cytoplasmic translation | GO:0002181 | 1.24E-104 |
| SFV | BP | ribosome biogenesis | GO:0042254 | 6.33E-72 |
| SFV | BP | rRNA processing | GO:0006364 | 1.85E-55 |
| SFV | BP | rRNA metabolic process | GO:0016072 | 7.08E-53 |
| SFV | BP | ribosomal large subunit biogenesis | GO:0042273 | 1.35E-50 |
| SFV | BP | ncRNA processing | GO:0034470 | 3.86E-46 |
| SFV | BP | ribosome assembly | GO:0042255 | 7.43E-34 |
| SFV | BP | non-membrane-bounded organelle assembly | GO:0140694 | 9.83E-27 |
| SFV | BP | maturation of LSU-rRNA | GO:0000470 | 2.81E-23 |
| SFV | BP | ribosomal large subunit assembly | GO:0000027 | 2.84E-22 |
| SFV | BP | ribosomal small subunit biogenesis | GO:0042274 | 4.33E-21 |
| SFV | BP | regulation of translation | GO:0006417 | 1.54E-20 |
| SFV | BP | regulation of cellular amide metabolic process | GO:0034248 | 1.65E-18 |
| SFV | BP | maturation of LSU-rRNA from tricistronic rRNA transcript (SSU-rRNA, 5.8S rRNA, LSU-rRNA) | GO:0000463 | 6.15E-16 |
| SFV | BP | negative regulation of cellular macromolecule biosynthetic process | GO:2000113 | 2.84E-12 |
| SFV | BP | negative regulation of translation | GO:0017148 | 9.96E-12 |
| SFV | BP | RNA localization | GO:0006403 | 1.31E-11 |
| SFV | BP | mRNA catabolic process | GO:0006402 | 3.67E-11 |
| SFV | BP | negative regulation of cellular amide metabolic process | GO:0034249 | 1.02E-10 |
| SFV | BP | RNA catabolic process | GO:0006401 | 1.48E-10 |
| SFV | BP | regulation of mRNA metabolic process | GO:1903311 | 2.98E-10 |
| SFV | BP | positive regulation of protein localization to chromosome, telomeric region | GO:1904816 | 4.75E-10 |
| SFV | BP | regulation of mRNA catabolic process | GO:0061013 | 1.03E-09 |
| SFV | BP | regulation of protein localization to chromosome, telomeric region | GO:1904814 | 2.33E-09 |
| SFV | BP | positive regulation of establishment of protein localization to telomere | GO:1904851 | 4.20E-09 |
| SFV | BP | positive regulation of translation | GO:0045727 | 4.25E-09 |
| SFV | BP | regulation of mRNA stability | GO:0043488 | 4.79E-09 |
| SFV | BP | positive regulation of protein localization to Cajal body | GO:1904871 | 1.14E-08 |
| SFV | BP | regulation of establishment of protein localization to telomere | GO:0070203 | 1.14E-08 |
| SFV | BP | regulation of protein localization to Cajal body | GO:1904869 | 1.14E-08 |
| SFV | BP | regulation of telomere maintenance | GO:0032204 | 1.18E-08 |
| SFV | BP | regulation of RNA stability | GO:0043487 | 1.53E-08 |
| SFV | BP | regulation of telomerase RNA localization to Cajal body | GO:1904872 | 1.54E-08 |
| SFV | BP | ribosomal small subunit assembly | GO:0000028 | 1.54E-08 |
| SFV | BP | RNA localization to Cajal body | GO:0090670 | 2.64E-08 |
| SFV | BP | RNA localization to nucleus | GO:0090685 | 2.64E-08 |
| SFV | BP | telomerase RNA localization | GO:0090672 | 2.64E-08 |
| SFV | BP | telomerase RNA localization to Cajal body | GO:0090671 | 2.64E-08 |
| SFV | BP | protein localization to Cajal body | GO:1904867 | 2.72E-08 |
| SFV | BP | protein localization to nuclear body | GO:1903405 | 2.72E-08 |
| SFV | BP | regulation of establishment of protein localization to chromosome | GO:0070202 | 2.72E-08 |
| SFV | BP | nucleobase-containing compound catabolic process | GO:0034655 | 3.77E-08 |
| SFV | BP | negative regulation of ubiquitin protein ligase activity | GO:1904667 | 5.84E-08 |
| SFV | BP | positive regulation of cellular amide metabolic process | GO:0034250 | 5.86E-08 |
| SFV | BP | protein localization to nucleoplasm | GO:1990173 | 1.16E-07 |
| SFV | BP | negative regulation of mRNA catabolic process | GO:1902373 | 2.08E-07 |
| SFV | BP | positive regulation of telomerase RNA localization to Cajal body | GO:1904874 | 2.15E-07 |
| SFV | BP | heterocycle catabolic process | GO:0046700 | 2.24E-07 |
| SFV | BP | regulation of RNA splicing | GO:0043484 | 2.71E-07 |
| SFV | BP | positive regulation of chromosome organization | GO:2001252 | 2.77E-07 |
| SFV | BP | cellular nitrogen compound catabolic process | GO:0044270 | 2.92E-07 |
| SFV | BP | maturation of 5.8S rRNA | GO:0000460 | 3.01E-07 |
| SFV | BP | maturation of SSU-rRNA | GO:0030490 | 3.08E-07 |
| SFV | BP | positive regulation of telomere maintenance | GO:0032206 | 4.65E-07 |
| SFV | BP | aromatic compound catabolic process | GO:0019439 | 5.57E-07 |
| SFV | BP | establishment of protein localization to telomere | GO:0070200 | 6.38E-07 |
| SFV | BP | regulation of telomere maintenance via telomerase | GO:0032210 | 6.91E-07 |
| SFV | BP | protein stabilization | GO:0050821 | 7.75E-07 |
| SFV | BP | mRNA stabilization | GO:0048255 | 1.21039E-06 |
| SFV | BP | negative regulation of RNA catabolic process | GO:1902369 | 1.42717E-06 |
| SFV | BP | signal transduction by p53 class mediator | GO:0072331 | 1.70321E-06 |
| SFV | BP | protein localization to chromosome, telomeric region | GO:0070198 | 2.45309E-06 |
| SFV | BP | negative regulation of ubiquitin-protein transferase activity | GO:0051444 | 2.47041E-06 |
| SFV | BP | regulation of signal transduction by p53 class mediator | GO:1901796 | 2.47901E-06 |
| SFV | BP | negative regulation of protein ubiquitination | GO:0031397 | 2.51148E-06 |
| SFV | BP | translational initiation | GO:0006413 | 2.94933E-06 |
| SFV | BP | regulation of telomere maintenance via telomere lengthening | GO:1904356 | 3.38033E-06 |
| SFV | BP | RNA stabilization | GO:0043489 | 5.42066E-06 |
| SFV | BP | positive regulation of telomere maintenance via telomerase | GO:0032212 | 5.48656E-06 |
| SFV | BP | regulation of chromosome organization | GO:0033044 | 7.80049E-06 |
| SFV | BP | telomere maintenance | GO:0000723 | 8.29332E-06 |
| SFV | BP | maturation of 5.8S rRNA from tricistronic rRNA transcript (SSU-rRNA, 5.8S rRNA, LSU-rRNA) | GO:0000466 | 1.06211E-05 |
| SFV | BP | negative regulation of cellular catabolic process | GO:0031330 | 1.12072E-05 |
| SFV | BP | telomere maintenance via telomerase | GO:0007004 | 1.13035E-05 |
| SFV | BP | maturation of SSU-rRNA from tricistronic rRNA transcript (SSU-rRNA, 5.8S rRNA, LSU-rRNA) | GO:0000462 | 1.13377E-05 |
| SFV | BP | positive regulation of telomere maintenance via telomere lengthening | GO:1904358 | 1.13377E-05 |
| SFV | BP | negative regulation of mRNA metabolic process | GO:1903312 | 1.28801E-05 |
| SFV | BP | negative regulation of protein modification by small protein conjugation or removal | GO:1903321 | 1.28801E-05 |
| SFV | BP | RNA-dependent DNA biosynthetic process | GO:0006278 | 1.30003E-05 |
| SFV | BP | regulation of protein stability | GO:0031647 | 1.36116E-05 |
| SFV | BP | establishment of protein localization to chromosome | GO:0070199 | 1.9809E-05 |
| SFV | BP | regulation of ubiquitin protein ligase activity | GO:1904666 | 1.9809E-05 |
| SFV | BP | regulation of DNA metabolic process | GO:0051052 | 3.62206E-05 |
| SFV | BP | negative regulation of catabolic process | GO:0009895 | 3.89488E-05 |
| SFV | BP | positive regulation of organelle organization | GO:0010638 | 5.55811E-05 |
| SFV | BP | telomere maintenance via telomere lengthening | GO:0010833 | 5.96104E-05 |
| SFV | BP | telomere organization | GO:0032200 | 7.29618E-05 |
| SFV | BP | regulation of protein modification by small protein conjugation or removal | GO:1903320 | 0.000164218 |
| SFV | BP | protein folding | GO:0006457 | 0.000242334 |
| SFV | BP | regulation of ubiquitin-protein transferase activity | GO:0051438 | 0.000348054 |
| SFV | BP | cleavage involved in rRNA processing | GO:0000469 | 0.000812804 |
| SFV | BP | stress granule assembly | GO:0034063 | 0.000812804 |
| SFV | BP | endonucleolytic cleavage involved in rRNA processing | GO:0000478 | 0.000843912 |
| SFV | BP | endonucleolytic cleavage of tricistronic rRNA transcript (SSU-rRNA, 5.8S rRNA, LSU-rRNA) | GO:0000479 | 0.000843912 |
| SFV | BP | ribosomal subunit export from nucleus | GO:0000054 | 0.000843912 |
| SFV | BP | ribosome localization | GO:0033750 | 0.000843912 |
| SFV | BP | positive regulation of DNA metabolic process | GO:0051054 | 0.000929909 |
| SFV | BP | protein localization to cytoplasmic stress granule | GO:1903608 | 0.000963162 |
| SFV | BP | positive regulation of protein localization to nucleus | GO:1900182 | 0.001004557 |
| SFV | BP | regulation of DNA biosynthetic process | GO:2000278 | 0.001013137 |
| SFV | BP | regulation of protein ubiquitination | GO:0031396 | 0.001160044 |
| SFV | BP | positive regulation of signal transduction by p53 class mediator | GO:1901798 | 0.00128039 |
| SFV | BP | protein localization to chromosome | GO:0034502 | 0.001947989 |
| SFV | BP | positive regulation of DNA biosynthetic process | GO:2000573 | 0.002024581 |
| SFV | BP | DNA biosynthetic process | GO:0071897 | 0.002140735 |
| SFV | BP | protein localization to nucleus | GO:0034504 | 0.002692778 |
| SFV | BP | nucleic acid transport | GO:0050657 | 0.003864727 |
| SFV | BP | RNA transport | GO:0050658 | 0.003864727 |
| SFV | BP | establishment of RNA localization | GO:0051236 | 0.004623444 |
| SFV | BP | actin filament organization | GO:0007015 | 0.00482687 |
| SFV | BP | alternative mRNA splicing, via spliceosome | GO:0000380 | 0.005136222 |
| SFV | BP | protein refolding | GO:0042026 | 0.005416204 |
| SFV | BP | regulation of translational initiation | GO:0006446 | 0.005652944 |
| SFV | BP | positive regulation of viral process | GO:0048524 | 0.007530109 |
| ZIKV | BP | respiratory electron transport chain | GO:0022904 | 2.70E-19 |
| ZIKV | BP | ATP synthesis coupled electron transport | GO:0042773 | 6.95E-19 |
| ZIKV | BP | mitochondrial ATP synthesis coupled electron transport | GO:0042775 | 6.95E-19 |
| ZIKV | BP | aerobic electron transport chain | GO:0019646 | 1.68E-18 |
| ZIKV | BP | mitochondrial electron transport, NADH to ubiquinone | GO:0006120 | 3.80E-18 |
| ZIKV | BP | mitochondrial respiratory chain complex assembly | GO:0033108 | 1.52E-17 |
| ZIKV | BP | oxidative phosphorylation | GO:0006119 | 8.19E-17 |
| ZIKV | BP | mitochondrial respiratory chain complex I assembly | GO:0032981 | 9.76E-17 |
| ZIKV | BP | NADH dehydrogenase complex assembly | GO:0010257 | 9.76E-17 |
| ZIKV | BP | aerobic respiration | GO:0009060 | 1.40E-16 |
| ZIKV | BP | cellular respiration | GO:0045333 | 4.71E-15 |
| ZIKV | BP | electron transport chain | GO:0022900 | 2.18E-14 |
| ZIKV | BP | energy derivation by oxidation of organic compounds | GO:0015980 | 6.55E-13 |
| ZIKV | BP | ATP metabolic process | GO:0046034 | 3.12E-12 |
| ZIKV | BP | generation of precursor metabolites and energy | GO:0006091 | 3.71E-09 |
| ZIKV | BP | inner mitochondrial membrane organization | GO:0007007 | 2.18E-05 |
| ZIKV | BP | ribosome biogenesis | GO:0042254 | 5.56262E-05 |
| ZIKV | BP | organelle fusion | GO:0048284 | 0.001073144 |
| ZIKV | BP | protein insertion into mitochondrial inner membrane | GO:0045039 | 0.001816545 |
| ZIKV | BP | vesicle fusion | GO:0006906 | 0.00249978 |
| ZIKV | BP | rRNA metabolic process | GO:0016072 | 0.003391859 |
| ZIKV | BP | organelle membrane fusion | GO:0090174 | 0.003635665 |
| ZIKV | BP | mRNA polyadenylation | GO:0006378 | 0.004057332 |
| ZIKV | BP | mitochondrial gene expression | GO:0140053 | 0.004357473 |
| ZIKV | BP | RNA polyadenylation | GO:0043631 | 0.004712176 |
| ZIKV | BP | mRNA 3'-end processing | GO:0031124 | 0.006171535 |
| ZIKV | BP | membrane fusion | GO:0061025 | 0.006926049 |
| ZIKV | BP | regulation of mRNA processing | GO:0050684 | 0.007491879 |
| ZIKV | BP | formation of cytoplasmic translation initiation complex | GO:0001732 | 0.009499933 |
| ZIKV | BP | organelle localization by membrane tethering | GO:0140056 | 0.009692004 |

**Supplementary References**

Batra, J., Hultquist, J. F., Liu, D., Shtanko, O., Von Dollen, J., Satkamp, L., et al. (2018). Protein Interaction Mapping Identifies RBBP6 as a Negative Regulator of Ebola Virus Replication. *Cell* 175, 1917-1930.e13. doi:10.1016/j.cell.2018.08.044.

Calderwood, M. A., Venkatesan, K., Xing, L., Chase, M. R., Vazquez, A., Holthaus, A. M., et al. (2007). Epstein-Barr virus and virus human protein interaction maps. *Proc Natl Acad Sci U S A* 104, 7606–7611. doi:10.1073/pnas.0702332104.

Contu, L., Balistreri, G., Domanski, M., Uldry, A.-C., and Mühlemann, O. (2021). Characterisation of the Semliki Forest Virus-host cell interactome reveals the viral capsid protein as an inhibitor of nonsense-mediated mRNA decay. *PLoS Pathog* 17, e1009603. doi:10.1371/journal.ppat.1009603.

de Chassey, B., Navratil, V., Tafforeau, L., Hiet, M. S., Aublin-Gex, A., Agaugué, S., et al. (2008). Hepatitis C virus infection protein network. *Mol Syst Biol* 4, 230. doi:10.1038/msb.2008.66.

Dolan, P. T., Zhang, C., Khadka, S., Arumugaswami, V., Vangeloff, A. D., Heaton, N. S., et al. (2013). Identification and comparative analysis of hepatitis C virus-host cell protein interactions. *Mol Biosyst* 9, 3199–3209. doi:10.1039/c3mb70343f.

Gordon, D. E., Hiatt, J., Bouhaddou, M., Rezelj, V. V., Ulferts, S., Braberg, H., et al. (2020a). Comparative host-coronavirus protein interaction networks reveal pan-viral disease mechanisms. *Science* 370, eabe9403. doi:10.1126/science.abe9403.

Gordon, D. E., Jang, G. M., Bouhaddou, M., Xu, J., Obernier, K., White, K. M., et al. (2020b). A SARS-CoV-2 protein interaction map reveals targets for drug repurposing. *Nature* 583, 459–468. doi:10.1038/s41586-020-2286-9.

Jäger, S., Cimermancic, P., Gulbahce, N., Johnson, J. R., McGovern, K. E., Clarke, S. C., et al. (2011). Global landscape of HIV-human protein complexes. *Nature* 481, 365–370. doi:10.1038/nature10719.

Khadka, S., Vangeloff, A. D., Zhang, C., Siddavatam, P., Heaton, N. S., Wang, L., et al. (2011). A physical interaction network of dengue virus and human proteins. *Mol Cell Proteomics* 10, M111.012187. doi:10.1074/mcp.M111.012187.

Li, J., Guo, M., Tian, X., Wang, X., Yang, X., Wu, P., et al. (2021). Virus-Host Interactome and Proteomic Survey Reveal Potential Virulence Factors Influencing SARS-CoV-2 Pathogenesis. *Med (N Y)* 2, 99-112.e7. doi:10.1016/j.medj.2020.07.002.

Shah, P. S., Link, N., Jang, G. M., Sharp, P. P., Zhu, T., Swaney, D. L., et al. (2018). Comparative Flavivirus-Host Protein Interaction Mapping Reveals Mechanisms of Dengue and Zika Virus Pathogenesis. *Cell* 175, 1931-1945.e18. doi:10.1016/j.cell.2018.11.028.

Shapira, S. D., Gat-Viks, I., Shum, B. O. V., Dricot, A., de Grace, M. M., Wu, L., et al. (2009). A physical and regulatory map of host-influenza interactions reveals pathways in H1N1 infection. *Cell* 139, 1255–1267. doi:10.1016/j.cell.2009.12.018.

Stukalov, A., Girault, V., Grass, V., Karayel, O., Bergant, V., Urban, C., et al. (2021). Multilevel proteomics reveals host perturbations by SARS-CoV-2 and SARS-CoV. *Nature* 594, 246–252. doi:10.1038/s41586-021-03493-4.

Wang, L., Fu, B., Li, W., Patil, G., Liu, L., Dorf, M. E., et al. (2017). Comparative influenza protein interactomes identify the role of plakophilin 2 in virus restriction. *Nat Commun* 8, 13876. doi:10.1038/ncomms13876.
